# Cooperation in spatial public good games depends on the locality effects of game, adaptation, and punishment

**DOI:** 10.1038/s41598-021-86668-3

**Published:** 2021-04-07

**Authors:** Isamu Okada, Hitoshi Yamamoto, Eizo Akiyama, Fujio Toriumi

**Affiliations:** 1 Faculty of Business Administration, Soka University, Hachioji, 192-8577 Japan; 2 Department of Information Systems and Operations, Vienna University of Economics and Business, Vienna, 1020 Austria; 3 Faculty of Business Administration, Rissho University, Tokyo, 141-8602 Japan; 4 Faculty of Engineering, Information and Systems, University of Tsukuba, Tsukuba, 305-8573 Japan; 5 Graduate School of Engineering, The University of Tokyo, Tokyo, 113-0033 Japan

**Keywords:** Social evolution, Applied mathematics, Computational science

## Abstract

Despite intensive studies on the evolution of cooperation in spatial public goods games, there have been few investigations into locality effects in interaction games, adaptation, and punishment. Here we analyze locality effects using an agent-based model of a regular graph. Our simulation shows that a situation containing a local game, local punishment, and global adaptation leads to the most robustly cooperative regime. Further, we show an interesting feature in local punishment. Previous studies showed that a local game and global adaptation are likely to generate cooperation. However, they did not consider punishment. We show that if local punishment is introduced in spatial public goods games, a situation satisfying either local game or local adaptation is likely to generate cooperation. We thus propose two principles. One is if interactions in games can be restricted locally, it is likely to generate cooperation independent of the interaction situations on punishment and adaptation. The other is if the games must be played globally, a cooperative regime requires both local punishment and local adaptation.

## Introduction

Even before it was selected as one of twenty-five big questions facing science in 2005^1^, numerous studies had tackled the fundamental puzzle of how cooperative behavior evolved. As a result of these academic efforts, several rationally understandable mechanisms have been proposed including kin-selection^2^, reciprocity^3^, structures^4^, and incentives^5^. However, many questions remain unanswered about the evolution of cooperation. Here, we focus on the combination problem between structures and incentives.

A public goods game (PGG)^6,7^ is a challenging social dilemma to resolve due to free riding issues, and thus has been dealt with in many theoretical studies. In this game, many players choose how many of their private tokens to put into a public pot, and all players equally receive the benefit (public good) made up of the tokens, irrespective of how many tokens they each put in. This game is regarded as an excellent analogy for numerous real-world issues including environmental protection, blood donation, and resource acquisition. Theory says that rationality in a situation without any specific conditions supports free-riding, and thus intensive studies have been exploring many types of conditions for generating and maintaining cooperation.

Although many analyses assume PGGs with well-mixed infinite populations because they are easy to analyze^8,9^, such homogeneity may not reflect real-world situations. Therefore, there have been many studies on special PGGs considering such a locality. In a review of spatial PGGs^4^, the results depended on not only the static structure but also group selection^10^, temporal network^11–13^, and population size^14^. When spatial structures are introduced into PGGs, players are more likely to cooperate, which is known as network reciprocity^15^. Many papers suggest that a heterogeneous network brings about cooperation in PGGs^16,17^ while a few papers show negative results^18–21^.

Although many studies have analyzed spatial PGGs, they almost all consider interactions of the players playing the games, and thus few papers have considered the locality of adaptive processes. For example, the players correspond their learning objects to their game partners. In another case, they learn from all players despite playing a spatial PGG. Some papers analyze this point^14,22^, but none has shown a case maintaining high cooperation in spatial PGGs.

Punishment is regarded as an excellent incentive for maintaining cooperation in PGGs, and thus it has been tested in not only theoretical analysis using evolutionary games^23–25^ but also empirical studies in social psychology^12,19,26,27^. A punishment system can be introduced into PGGs in several ways. The typical punishment system is peer-punishment, in which non-contributors in a PGG are punished by the other players^5,28,29^. Peer-punishment itself is also a kind of public good, and thus a theoretical analysis has shown that such a peer-punishment system is evolutionary unstable^5,30^. To overcome this instability, a pool punishment system was proposed^30^, in which punishment costs for maintaining an institutional punishment system (a police officer) must be paid before playing the game, and two types of players (non-cooperators in the PGG and free-riders for punishment costs) are punished by the system. However, several studies have found that the pool-punishment system is inefficient because players must pay the cost even if the non-cooperators are eliminated^31–33^. The conflict between peer punishment and pool punishment continues because of a trade-off between stability and efficiency.

While theoretical studies on punishment introduced in spatial PGGs reveal that it is likely to lead to cooperation^34^, almost all previous studies assume that peer-punishment is allowed among game partners^35–38^. Therefore, few studies have analyzed cases in which the punishment scope is independent of the interaction scope of the game.

Since there has been no systematic analysis of whether the scope of the interaction is global or local, it is not unnatural that the interaction scope of the game does not match that of the learning target or the scope of peer-punishment. Here, we analyze the locality effects of interactions in games, adaptation, and punishment in spatial PGGs. To do so, we analyze a model of players located on a regular network using agent-based simulations. Our model focuses on the locality effects using expected payoffs rather than actual payoffs. In general, the impact of randomness should be carefully considered when adopting an agent-based model. Including the adaptation process in an agent-based model often requires two time scales: play time and adaptation time. One period of adaptation often consists of millions of game plays, as players need to accumulate payoffs by playing multiple times to minimize the negative effects of randomness. Since the purpose of this study is to clarify the effect of locality, we believe that maximally eliminating the effect of randomness will clearly show the simulation results using the expected payoff. Our model uses randomness only when selecting the target player in the adaptation process.

## Methods

First, we define four types of players in the model: cooperative punisher (CP), cooperative non-punisher (CN), non-cooperative punisher (DP), and non-cooperative non-punisher (DN). A cooperator always contributes when playing a PGG while a non-cooperator never does. We do not consider any type of error in this version. A punisher always punishes non-cooperators when the punisher sees them while a non-punisher never does. We do not consider anti-social punishment^12,39^ in this version.

To simply express the three types of locality, *N* players are located on a one-dimensional regular graph of degree 2. To consider the locality of each player, one’s neighbors are defined as players within *n*/2 hops excluding oneself. Note that *n* should be an even number. For example, if $n=4$, every player recognizes one’s neighbors as (1) two players directly connected to oneself and (2) two more players directly connected to those directly connected to oneself. Compared with a scale-free network or a small-world network, all players in our model maintain the network’s homogeneity.

We explain how to introduce three types of locality with respect to interactions in the game, adaptation, and punishment. In the interaction in the game, first, we introduce a parameter, $g \in [0,1]$. We assume this parameter to be the degree of social (im)mobility. All players play a PGG with $n+1$ players including oneself. The other members are randomly chosen from the two pools of players: a pool consisting of one’s *n* neighbors and a pool consisting of all $N-1$ players. The ratio of the quantity (we allow non-integer numbers) chosen from each pool is $g:1-g$. Second, we introduce a parameter, $p \in [0,1]$ for dealing with a locality of punishment. With a probability, *p*, a player (if one is a punisher) looks at *n* players that are randomly chosen among all $N-1$ players excluding oneself and punishes non-cooperators among those *n* players. Otherwise, the player looks at *n* neighbors and punishes non-cooperators among them. Finally, we introduce a parameter, $a \in [0,1]$. With a probability, *a*, a player chooses a target player for one’s learning among all $N-1$ players excluding oneself. Otherwise, the player chooses a target player among one’s *n* neighbors.

When playing a PGG, if a player gives a token to a public pod, one must pay a cost, *c*. Otherwise, no cost is paid. A benefit of a token is set to *b*, and thus if the number of contributors in a game is $n_c$ while the number of all players of the game is $n_a$, then each player is given $b n_c / n_a$ as one’s benefit. After playing a PGG, all punishers are given an opportunity to punish. If player *X* punishes player *Y*, *Y* loses *f* as a fine while *X* pays *s* as a sanction for the punishment.

Following the law of large numbers, each player’s payoff per game playing over a sufficiently long time corresponds to the expected value, and thus we use one’s expected payoff. Therefore, player *i*’s payoff of each type is defined as

1 $$\begin{aligned} \Pi _i^{CP}&= \pi _{C_i} - \pi _{P_i} \end{aligned}$$

2 $$\begin{aligned} \Pi _i^{CN}&= \pi _{C_i} \end{aligned}$$

3 $$\begin{aligned} \Pi _i^{DP}&= \pi _{D_i} - \pi _{P_i} - \pi _{F_i} \end{aligned}$$

4 $$\begin{aligned} \Pi _i^{DN}&= \pi _{D_i} - \pi _{F_i} \end{aligned}$$

where

5 $$\begin{aligned} \pi _{C_i}&= b [n \{ g N_C^{-i} + (1-g) n^i_C \} + 1]/(n+1) - c \end{aligned}$$

6 $$\begin{aligned} \pi _{D_i}&= b n \{ g N_C^{-i} + (1-g) n^i_C \} /(n+1) \end{aligned}$$

7 $$\begin{aligned} \pi _{P_i}&= s \{ p (1 - N_C^{-i}) + (1-p) (1- n^i_C) \} \end{aligned}$$

8 $$\begin{aligned} \pi _{F_i}&= f \{ p N_P^{-i} + (1-p) n^i_P \} \end{aligned}$$

and $N_C^{-i}$, $n^i_C$, $N_P^{-i}$, and $n^i_P$ are respectively the ratio of cooperators among all $N-1$ players excluding oneself (*i*), the ratio of cooperators among player *i*’s neighbors, the ratio of punishers among all $N-1$ players excluding oneself (*i*), and the ratio of punishers among player *i*’s neighbors.

Every player has a chance to update one’s type through an adaptation process. In the process, every player (*X*) chooses another player (*Y*) using the selection rule explained in the part of the definition of *a*. We assume that all players simultaneously have an opportunity to revise their types, and we refer to the period until the end of this adaptation as a generation. Player *X* changes one’s strategy to *Y*’s if and only if *Y*’s payoff is greater than *X*’s. Moreover, we introduce a mutation rate, $\mu$, to keep the diversity of player types. With a probability, $\mu$, an agent uses one of four types randomly in each adaptive process.

In each simulation, the parameters on locality (*g*, *p*, *a*) are given as constants. In an initial setting of a simulation, all players on a regular graph are set to one of the four types randomly. Then each player’s expected payoff is calculated and their types are updated in the adaptation process. This procedure is repeated and the performances are observed.

## Results

**Figure 1:**
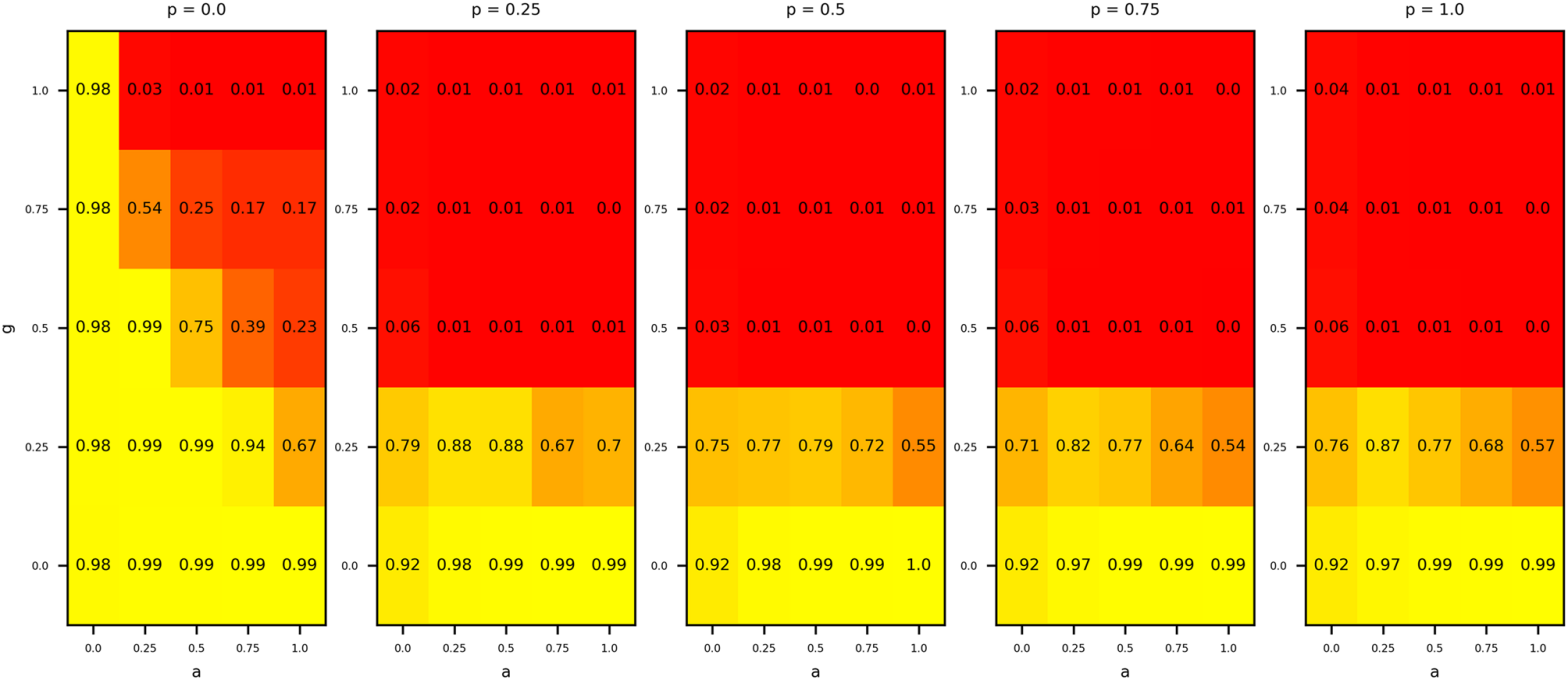
Average cooperation rates in 100 trails after 100 generations. From the left, each panel is a case of $p=0, 0.25, 0.5, 0.75, 1$, respectively. In each panel, the horizontal and vertical axes represent the values of *a* and *g*, respectively. The panel is a heat map, and the values shown are the average cooperation rates. Parameters are $(N,n)=(100,4)$, $(b,c,f,s)=(2,1,6,3)$, and $\mu =1\%$. This image is made by using Python 3.7.2 (www.python.org).

Our simulation shows the locality effects on cooperation. In Fig. 1, it is clear that the more local the game, the more cooperative the regime. Among three elements, this effect matters. This is because when $g=0$, all cases maintain cooperative regimes regardless of the values of the other two parameters (*p*, *a*). Moreover, when $g=0.5$ or more, all cases except for $p=0$ collapse the cooperative regimes. Second, considering the values of *p*, the regime changes drastically depending on whether *p* is almost zero or not (see Fig. S1 in Supplementary Information). When $p=0$, it is possible to maintain cooperation even if *g* is large. Otherwise, the cooperative regime requires *g* to be small. Finally, we consider the parameter *a*. We must split it into two cases: *g* is almost zero or not (see Fig. S2 in Supplementary Information). If $g=0$, the greater the value of *a*, the more cooperative the regime. On the other hand, if at least $g \ge 0.25$, the greater the value of *a*, the less cooperative the regime. This result suggests *a* needs to be analyzed in more detail.

**Figure 2:**
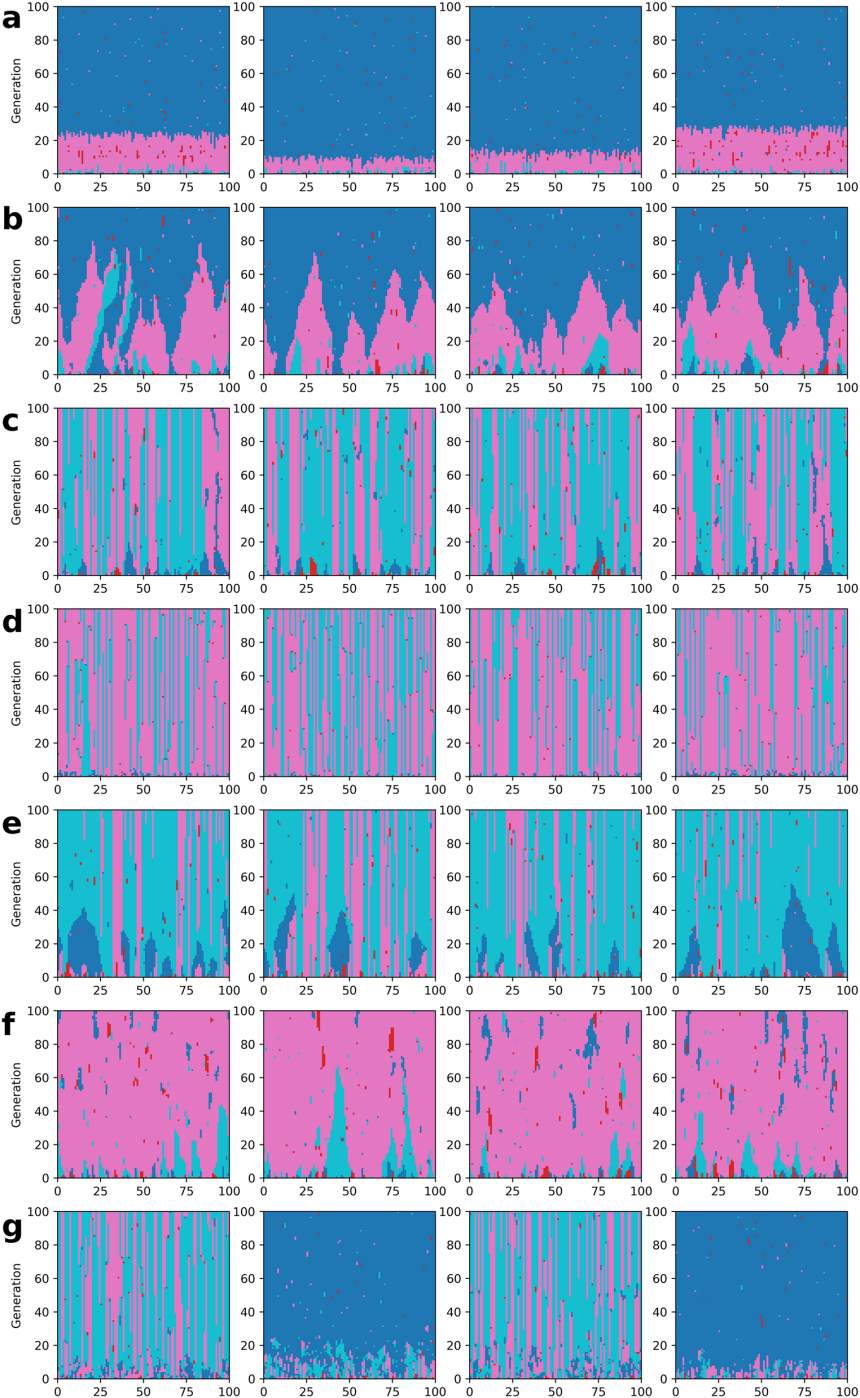
Transition diagram of strategy in 100 generation. From the top, each row is a case of different values of (*g*, *p*, *a*): **(a)** (1, 1, 1), **(b)** (0.5, 0.25, 0), **(c)** (0, 0, 0), **(d)** (0, 0, 1), **(e)** (1, 0, 0), **(f)** (0, 1, 0) and **(g)** (0.5, 0, 0.5). In each row, 4 trials are shown with different random seeds on the same parameter set. In each panel, all 100 agents are in a queue of a row. The horizontal axis corresponds to agent’s id. Agents with consecutive numbers are next to each other on the regular network. For example, the agents next to an agent having $id=24$ are $id=23$ and $id=25$. Agents of $id=99$ and $id=0$ are next to each other. In each graph, the lowest row shows their strategies at the first generation. Also, the second row shows those at the second generation, while the most top row shows those at the 100-th generation. An agent whose strategy is *CP* is colored in light blue, *CN* in pink, *DP* in red, and *DN* in dark blue. Parameters are $(N,n)=(100,4)$, $(b,c,f,s)=(2,1,6,3)$, and $\mu =1\%$. This image is made by using Python 3.7.2 (www.python.org).

Figure 2 shows transitions on players’ strategies in specific cases. First is panel (a). The CP players assigned randomly in the initial state are soon eliminated by the CN players, as taught in standard game theory (punishing itself is regarded as a public good). However, the CN players who dominate the population temporally are also beaten out by the perfect free riders (DN) within most 30 generations. This result is consistent with many previous papers showing that neither punishment nor cooperation works in a well-mixed population without an interaction structure^40^. Panel (b) shows a different case but corresponds to panel (a) qualitatively while its transition speed is slower. Even if the locality of adaptation is completely executed, it is not likely to generate cooperation when the locality of the game and punishment is insufficient. This result indicates that two local interaction structures in game and punishment are necessary to maintain cooperative regimes.

Panels (c) and (d) are the cases of $(g,p)=(0,0)$, which means there is a strong locality for both the game and the punishment. Both panels have a vertically-striped pattern of CP and CN that reflects the trait of locality. Due to this trait, the alliance between CPs is possible, and thus the presence of CPs prevents defectors (both DP and DN) from invading. We consider the reason a DN cannot spread in a population of CNs to be that interaction partners of the two neighbor players do not completely correspond. Let us consider the case of “C C C C D D D” where $n=4$. The third player is CN and one’s neighbors are CCCD, and thus the expected payoff is $4b/5 - c$. The fifth player is DN and one’s neighbors are CCDD, and thus the expected payoff is 2*b*/5. The payoff of the third player is greater than that of the fifth if $2b > 5c$. This is why if the fifth player chooses the third in an adaptation process, it is possible to change from D to C.

Comparing these two cases, panel (d) is more robust than panel (c) because DN mutants more easily spread in (c) than in (d). This mechanism is related to an interaction scope with respect to learning. If the learning scope is narrow ((c) $a=0$), free riding by a DN player attracts the attention of its neighbors and thus it helps DN spreading. Otherwise ((d) $a=1$), free riding by a DN player decreases its neighbor’s payoffs, and thus a far player surrounded by cooperators attracts attention. Therefore, a global adaptation process is effective if a regime is cooperative. This reasoning is consistent with Fig. 1.

When we look at panel (e), it is easy to understand that the locality of game size is essential to keep cooperative regimes. Comparing it with panel (c), the group size of DN can easily become big. This is because a free rider barely affects its neighbor’s payoffs, and thus its local adaptation process chooses such a free rider. However, such free riders may go extinct sooner or later because the local punishment ($p=0$) works. If a CP player and a DN player are placed adjacently, the DN player is directly punished by the CP player while the defection barely affects the CP player, and thus CP can invade the population of DN players. Comparing it with the case of $(g,p,a)=(1,1,0)$ in Fig. 1, such a local punishment is necessary to maintain cooperative regimes.

Next, we consider the effect of the locality of punishment using panel (f). When there is no locality with respect to punishment ($p=1$), punishers make much effort to punish all defectors of the population. That is why the population of punishers tends to diminish except for the mutation process. Such a cooperative regime is very fragile. In the case of $(g,p,a)=(0,1,0)$ in Fig. 1 and Fig. S3 in Supplementary Information, $8\%$ of the players are defectors on average. However, if a regime is cooperative, a global adaptation works for maintaining the regime. Suppressing defectors decreases the opportunity to destroy a cooperative regime.

Finally, we analyze the effect of the adaptation process when $\hbox {g} > 0$ using panel (g). Comparing it with panel (c), the colony size of DN tends to grow due to the weak locality in the game, as previously discussed in the case of panel (e). This tendency and the global adaptation process help to imitate defectors because defectors gain greater payoffs than cooperators generally. As a result, cooperative regimes can collapse or be maintained due to randomness. If a player chooses a neighbor, the cooperative regime can be maintained. In contrast, if a player selects a distant player and the distant player adopts a DN strategy, the regime collapses. See Fig. S4 in SI for more information. This result suggests an interesting feature in the adaptation process. When the cooperative regime is basically maintained, the global adaptation process spreads the well-behaved CP in the regime. On the other hand, if the cooperative regimes are fragile, the global adaptation process helps the selfish DNs to expand in the regime.

To sum up the results, the case most likely to generate cooperation is $(g,p,a)=(0,0,1)$ (panel (d) in Fig. 2). Defectors are likely to invade if the locality of the game loosens (panel (e)), while punishers become rare if the locality of the punishment loosens (panel (f)). If those two localities can maintain cooperative regimes, the globality of adaptation can increase their robustness.

## Discussion

We conducted agent-based simulations to analyze the effects of the degree of locality on interactions of the game, punishment, and adaptive process in the evolution of cooperation in spatial PGGs. Our exhaustive analysis clarifies what features of those three locality effects are likely to generate cooperation. Roughly speaking, if an interaction of games can be restricted locally, it is likely to generate cooperation independent of the interaction situations in punishment and adaptation. If the games are played globally, a cooperative regime requires both local punishment and local adaptation.

Our simulation shows an interesting feature in local punishment. Previous studies showed that local game and global adaptation are likely to generate cooperation^14,22^. However, they did not consider punishment. We show that if the local punishment is introduced, a situation satisfying either local game or local adaptation is likely to generate cooperation. Cooperative behavior in PGGs and punishing behavior in punishment systems are both tools for maintaining cooperation. If both fail, cooperation will collapse. Cooperation can be maintained more robustly with two tools than with only one.

In general, punishment systems are effective for maintaining cooperation in PGGs. However, according to our analysis, the local interactions in game and punishment are likely to generate cooperation. This reaffirms that punishment as well as games has the nature of a public good. Moreover, punishment is a more naive public good, and if the effects of punishment are attenuated by globality, punishment cannot be used to curb surrounding defectors and is more likely to be eliminated.

While we clarify the effects of local punishment on the cooperation, we must consider a way to enhance punishment behaviors as a public good. Cong et al.(2017) simulated a case in which punishers were allowed to have a runaway option if many defectors were in their neighbors. According to their results, such an option helps the adaptive survival of punishers. While the current version of our model does not give a player the freedom to move, such an extension should be analyzed in future work.

This study has possible extensions. A comprehensive analysis is required to determine the cost-benefit effects of games, the efficiency of punishment, and the cost ratio of cooperation and punishment. For example, if the cost of punishment and fines are relatively high, a cooperative regime may be able to be maintained with only a few punishers who can come in via mutations. In the current version, our model uses a regular graph to eliminate network heterogeneity. We estimate that the current results may be sensitive to and dependent on the network structure, and thus we are also interested in the performance in island models and other heterogeneous network structures.

This paper simulates an extremely limited version of the network structure, so the robustness of the results should be carefully considered. In future works, the model should be extended to 2-dimentional networks such as square lattices. The stochasticity in a payoff, which is absent due to the setting of expected payoff in the current version, can also be considered. Population size is not expected to affect cooperation. Rather, we presume that the network structure and the size of the neighborhood are especially important factors for the regime. Future work will investigate the validity of our estimate.

## Supplementary information

Supplementary figures
