# Supplementary material for: Cooperation in spatial public good games depends on the locality effects of game, adaptation, and punishment
Source: Sci Rep. 2021 Apr 7;11:7642. doi: 10.1038/s41598-021-86668-3 (PMC8026997; doi:10.1038/s41598-021-86668-3)
Supplement: Supplementary file 1 — Supplementary figures [file 41598_2021_86668_MOESM1_ESM.pdf]

Supplementary Information

for

Cooperation in spatial public good games depends on the locality effects of game, adaptation, and punishment.

Isamu Okada, Hitoshi Yamamoto, Eizo Akiyama, Fujio Toriumi

Figures

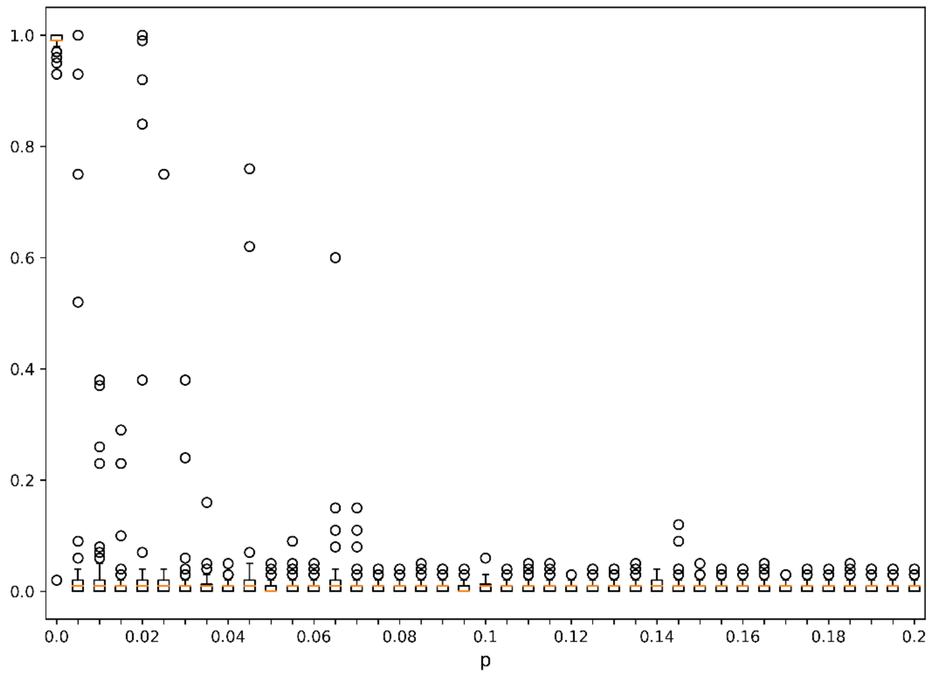

Fig. S1: A boxplot on cooperation rates in 100 trials after 100 generations with respect to the locality effect of punishment. This figure is for understanding details of the parameters (g,a) = (0.5, 0.25) in Fig. 1 of the main text. Note that this graph covers performances where the range of p is between 0 and 0.2. While the median of the cooperation rates when p = 0 is almost one, that when p = 0.01 goes to almost zero. This image is made by using Python 3.7.2 (www.python.org).

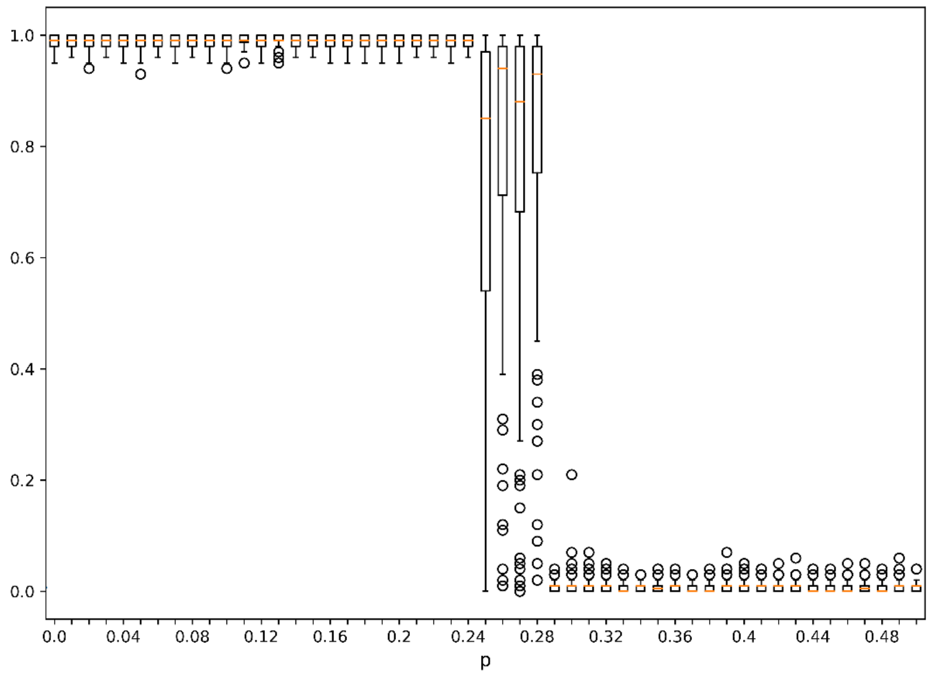

Fig. S2: A boxplot on cooperation rates in 100 trials after 100 generations with respect to the locality effect of game. This figure shows supporting details of the parameters (p,a) = (0.5,0.5) in Fig. 1 of the main text. Note that this graph covers performances where the range of g is between 0 and 0.5. The regime change occurs when a value of g is between 0.25 and 0.3. This image is made by using Python 3.7.2 (www.python.org).

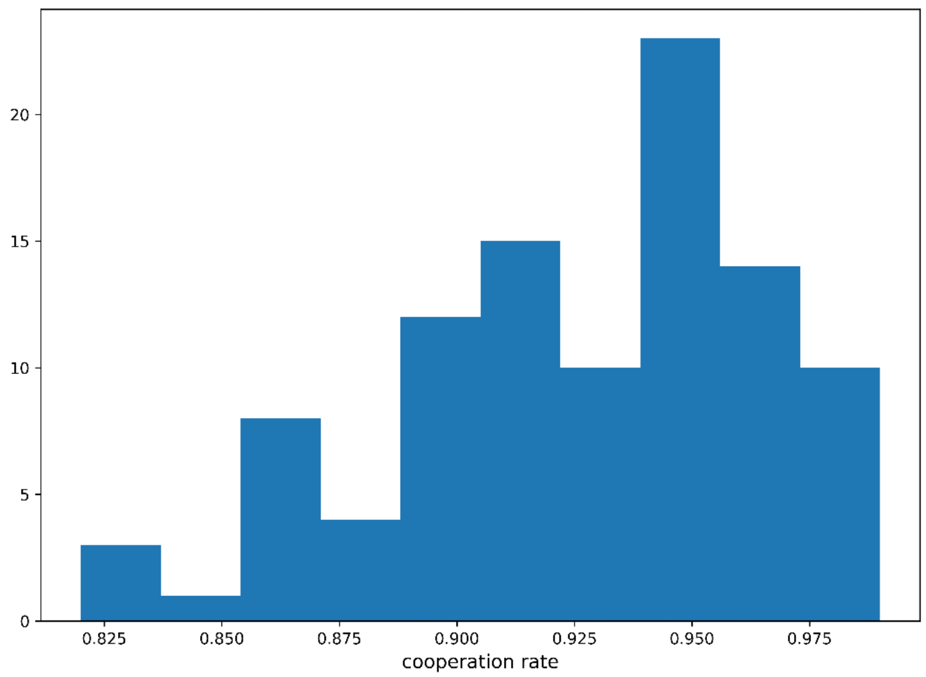

Fig. S3: A histogram of cooperation rates in 100 trials after 100 generations in the case of (g,p,a)=(0,1,0). This figure shows supporting details for Fig. 2(f) of the main text. This graph clearly shows that the rate of cooperators in all simulations stays 80% or more while a few defectors survive. This image is made by using Python 3.7.2 (www.python.org).

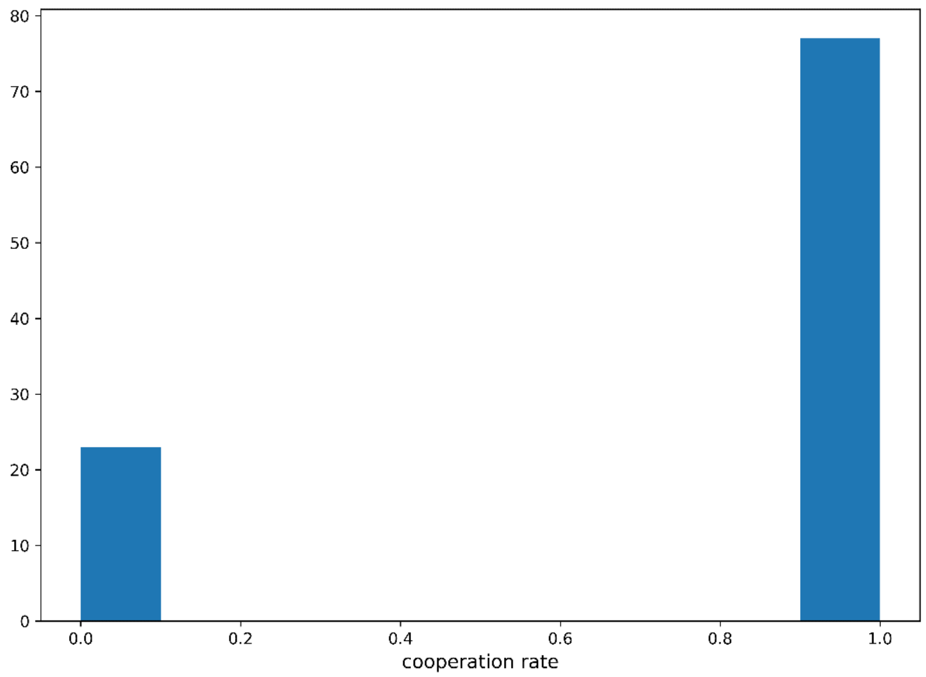

Fig. S4: A histogram of cooperation rates in 100 trials after 100 generations in the case of (g,p,a)=(0.5,0,0.5). This figure shows supporting details for Fig. 2(g) of the main text. This graph clearly shows that the simulation results after 100 generations are either cooperative (cooperative rate exceeds 0.9) or non-cooperative (cooperation rate is less than 0.1). This image is made by using Python 3.7.2 (www.python.org).
